# Differences in mental illness stigma by disorder and gender: Population-based vignette randomized experiment in rural Uganda

**DOI:** 10.1371/journal.pmen.0000069

**Published:** 2024-06-21

**Authors:** Yang Jae Lee, Ryan Christ, Rita Mbabazi, Jackson Dabagia, Alison Prendergast, Jason Wykoff, Samhitha Dasari, Dylan Safai, Shakira Nakaweesi, Swaib Rashid Aturinde, Michael Galvin, Dickens Akena, Scholastic Ashaba, Peter Waiswa, Robert Rosenheck, Alexander C. Tsai

**Affiliations:** 1 Department of Psychiatry, Yale University School of Medicine, New Haven, Connecticut, United States of America; 2 Empower Through Health, St. Louis, Missouri, United States of America; 3 Department of Genomics, Yale University School of Medicine, New Haven, Connecticut, United States of America; 4 College of Arts and Sciences, University of Michigan, Ann Arbor, Michigan, United States of America; 5 College of Arts and Sciences, University of Georgia, Athens, Georgia, United States of America; 6 Williams College, Williamstown, Massachusetts, United States of America; 7 Department of Psychiatry, Boston Medical Center, Boston, Massachusetts, United States of America; 8 Department of Psychiatry, Makerere University College of Health Sciences, Kampala, Uganda; 9 Department of Psychiatry, Mbarara University of Science and Technology, Mbarara, Uganda; 10 Department of Psychiatry, Makerere University School of Public Health, Kampala, Uganda; 11 Center for Global Health and Morgan Institute, Massachusetts General Hospital, Boston, Massachusetts, United States of America; 12 Department of Psychiatry, Harvard Medical School, Boston, Massachusetts, United States of America; Arak University of Medical Sciences, IRAN, ISLAMIC REPUBLIC OF

## Abstract

Understanding and eliminating mental illness stigma is crucial for improving population mental health. In many settings, this stigma is gendered, from the perspectives of both the stigmatized and the stigmatizers. We aimed to find the differences in the level of stigma across different mental disorders while considering the gender of the study participants as well as the gender of the people depicted in the vignettes. This was a population-based, experimental vignette study conducted in Buyende District of Eastern Uganda in 2023. We created 8 vignettes describing both men and women with alcohol use disorder, major depressive disorder, generalized anxiety disorder, and schizophrenia consistent with DSM-5 criteria. Participants from 20 villages in rural Buyende District of Uganda (N = 379) were first read a randomly selected vignette and administered a survey eliciting their attitudes (Personal Acceptance Scale [PAS] and Broad Acceptance Scale [BAS]) towards the person depicted in the vignette. We used analysis of variance (ANOVA) with Bonferroni-adjusted, empirical p-values to compare levels of acceptance across disorders and genders. Attitudes towards people with mental illness, as measured by the PAS, varied across different mental disorders (p = 0.002). In pairwise mean comparisons, the greater acceptance of anxiety disorder vs. schizophrenia was statistically significant (Mean [SD] PAS: 2.91 [3.15] vs 1.62 [1.95], p = 0.008). Secondary analyses examining differences in acceptance across gender combinations within mental disorders showed that PAS varied across gender combinations for depression (p = 0.017), suggesting that acceptance is higher for women with depression than men with depression. In this population-based vignette study from rural Uganda, we found that people with schizophrenia were less accepted compared to people with anxiety disorders. We also found that there was greater acceptance of women with depression than men with depression. Anti-stigma initiatives may need to be targeted to specific disorders and genders.

**Trial registration:** The experimental procedures for this study were registered with ClinicalTrials.gov as “Survey Experiment to Estimate Level of Mental Illness Stigma Based on Condition and Gender” (NCT 06279962).

## Background

Stigma towards mental illness is a worldwide phenomenon that leads to substantial psychological distress, inhibition of help seeking behavior, limited provision of treatment resources, and societal ostracism especially in low and middle income countries (LMICs) [1–5]. In many cases, individuals with mental health disorders are subjected to abuse, isolation, and discrimination [2,6,7]. In Uganda, only 15% of individuals with mental illnesses receive any mental health treatment [2]. This low rate of treatment adherence can be attributed to multiple factors, including economic constraints, stigma, and limited healthcare resources [8]. While it is noted that merely 1% of Uganda’s healthcare budget is dedicated to mental health services, this statistic is not an outlier, with 24.4% of countries out of 78 studied spending below 1% of health expenditure on mental health [9]. Uganda, like many other countries, suffers from a severe shortage of trained specialists and limited access to effective treatments [2,10].

Given the negative impacts of stigma on people with mental illness and their families, treatment availability and healthcare seeking behavior, understanding and reducing mental illness stigma is a public health priority [11,12]. However, despite considerably heterogeneous presentations of mental illness, studies of stigma often group different mental disorders together and design anti-stigma interventions towards people with mental illness in general, rather than for specific disorders [13]. Some research in high-income countries (HICs) has attempted to address this knowledge gap, showing that there is more stigma attached to schizophrenia compared to depression or anxiety, although there is significant variation across life domains and across countries [3,4,14,15].

Another facet of mental illness stigma that is poorly understood is the influence of gender on the stigma towards people with mental disorders. Research conducted in HICs has indicated that individuals with mental illnesses whose symptoms align with gender-based stereotypes are more susceptible to experiencing stigma [16]. For instance, a man with schizophrenia is portrayed as potentially violent and may encounter heightened stigmatization compared to a woman with the same condition [16]. A study examining mental health stigma and substance abuse disorders in the United States, which analyzed factors like gender, ethnicity, and education, found that women exhibit a lower tendency to endorse discriminatory behaviors towards individuals with psychiatric disorders when compared to men [6,17]. In India, a lower middle-income country with clearly demarcated gender roles, individuals with schizophrenia experienced stigma most strongly in relation to their failure to meet gendered norms–for example, men in regards to employment and women in relation to marriage and childbirth [18]. However, literature on the topic has been relatively scant, and no studies have assessed differential levels of mental illness stigma by gender in sub-Saharan Africa.

To address this gap in the literature, we investigated differences in the level of societal and public stigma across different mental disorders while considering the gender of the study participants as well as the gender of the people depicted in the vignettes. The four mental disorders addressed in this study include major depressive disorder, generalized anxiety disorder, alcohol use disorder, and schizophrenia, as these disorders account for some of the highest disability-adjusted life years among mental illnesses globally [19]. Findings from this study could add nuance to the understanding of mental health stigma in LMICs and suggest specific differences in focus for future anti-stigma interventions.

## Methods

### Ethics statement

Consent forms were translated from English to Lusoga by native Lusoga speakers, and a Lusoga-speaking psychiatric clinical officer back-translated them to English to verify their fidelity to the original document. Prospective study participants were approached by research assistants who presented a consent form outlining the study’s purpose, potential risks, and discomforts, seeking written consent at their homes. Participants who could not read/write/sign their name were permitted to indicate consent with a fingerprint in the presence of a witness who signed the consent form. Surveys were administered in Lusoga utilizing a printed-out questionnaire. After surveys were administered in the field, data were entered into KoboToolbox (v2021.2.4, Kobo Inc., Cambridge, Mass.) on an encrypted mobile device.

All study procedures were conducted in accordance with relevant guidelines and regulations. This project was approved by the Institutional Review Boards of The AIDS Support Organization, Uganda (TASO-2023-222) and Yale University (2000034605). We were cleared to conduct the study by the Uganda National Council of Science and Technology (SS1860ES). The study was registered on clinicaltrials.gov (NCT06279962). Due to an administrative error, study was not entered and released on ClinicalTrials.gov until February 2024.

### Study site

This research was conducted in the Irundu, Kagulu, and Gumpi sub-counties of Buyende District, a rural area situated in the Busoga region of eastern Uganda with an estimated population of 468,400 [20]. Buyende District was selected for this study because it is representative of many rural areas in Uganda. Almost half (45%) of individuals aged 18 and older cannot read/write, and most of the working population are engaged in subsistence farming [20].

### Selection of participants

A numbered list of all villages in the Irundu, Kagulu, and Gumpi sub-counties was used to select 20 villages utilizing a random sampling design through a random number generator. All individuals in the selected study site over 18 years of age and able to provide informed consent were eligible for the study. Individuals under the age of 18 or who could not give consent to participate in this study were excluded. After obtaining a household census from a community health worker in each village, 20 households were selected from each village utilizing a random number generator. Finally, all adult members of the selected households were assigned a random number, and a random number generator was used to identify one adult member to be interviewed for this study. The final sample included 379 study participants.

### Study procedures

Eight vignettes, each describing a man or woman with a specific mental disorder, were assigned a number between one and eight and then selected for presentation to respondents using a random number generator. Research assistants utilized the random number generator After the vignette was read to participants, they were asked to complete a structured questionnaire eliciting their attitudes towards the individual described in the vignette. Data collection for the study took place between July 2023 and September 2023.

The vignettes depicting generalized anxiety disorder, schizophrenia, alcohol use disorder, and depression were read to participants and were adapted from a previously published study conducted in southwestern Uganda [21], the vignettes for which were themselves adapted from a study conducted in the U.S [15]. [S1 Text] Vignettes on anxiety and alcohol use disorder were developed through a collaborative effort from Ugandan and American researchers. The four vignettes then were modified to reflect both sexes so that we could assess the extent to which the sex of the individual depicted in the vignette influenced study participants’ responses. To ensure their relevance and accuracy for the current Ugandan context, these vignettes underwent a detailed adaptation process. This included modifications by a collaborative team of Ugandan and American researchers, which included psychiatrists. These experts ensured that the vignettes were consistent with local communication styles and cultural nuances. The vignettes were then pre-tested on a small sample of the local population to ensure they were understandable and comprehensible. Thus, these procedures yielded vignettes with a high degree of acceptability, face validity, and content validity. There were 8 vignettes total.

After the vignette was read aloud to the participants, they were administered a brief questionnaire. This questionnaire included two sub-scales: the Personal Acceptance Scale (9 items) and the Broad Acceptance Scale (9 items). [S2 Text] Both scales were adapted from a study examining attitudes and beliefs about mental illness in Nigeria [22,23], and previously utilized in Uganda [24,25]. The original questionnaire utilized in the Nigerian study modified items taken from the Fear and Behavioral Intentions towards the Mentally Ill scale [26], selected items from the Community Attitudes to Mental Illness scale [27], and a modified version of a questionnaire developed for the World Psychiatric Association’s Program to Reduce Stigma and Discrimination [28].

Items included in the Personal Acceptance Scale measure desire for personal distance from those with mental illness (e.g., “I would not want to live next door to this person”) and can be conceptualized as a social distancing scale [29]. The items included in the Broad Acceptance Scale measure both personal attitudes towards people with mental illness (e.g., “People with the condition depicted are a public nuisance”) as well as personal attitudes about what a society should do for those with mental illness (e.g., “Increased spending on services for people like this is a waste of money”). Thus, both scales measure aspects of public stigma, the “negative attitudes, beliefs, and behaviors held within a community” (p.22) [30] against individuals with mental illness.

Each item was scored on a dichotomous scale (yes/no), with each scale ranging in value from a minimum of 0 to a maximum of 9. Five items were reverse coded such that higher scale values represented more accepting (less stigmatizing) attitudes.

### Statistical analysis

The analysis plan was executed independently for both outcomes, PAS and BAS. To test for differences in acceptance across four psychiatric conditions, we fitted a linear mixed model. To account for potential gendered patterning in stigma, this model included four fixed effects (including the intercept) to allow for different mean outcomes within each of the four possible gender combinations: a female study participant exposed to a vignette featuring a woman; a female participant exposed to a vignette featuring a man; a male participant exposed to a vignette featuring a woman; and a male participant exposed to a vignette featuring a man. The model also included a village-level random effect.

We then fitted a similar regression model with 16, rather than 4, fixed effects. The 16 fixed effects were generated by fully interacting the four vignette conditions—depression, schizophrenia, anxiety, and alcohol use disorder—with the four possible gender combinations. This allowed the alternative model to capture differences in acceptance across the four conditions, allowing for gendered patterning and village effects. We compared this model to the model without interaction terms using a likelihood ratio test. For robustness against model misspecification, we reported an empirical p-value based on 100,000 permutations of the outcome vector. These permutations were done independently within subgroups determined by the intersection of village, sex of the survey respondent, and the sex of the individual portrayed in the vignette. In other words, samples had to match on these three variables for their corresponding outcomes to be permuted.

To further explicate any observed differences between conditions identified in our primary analysis, we then undertook a secondary analysis testing for equivalence in stigma between each pair of mental disorders, adjusting for gendered patterning and village effects. We started with the regression model with 16 fixed effects from our primary analysis. For each of the 6 possible pairs among the 4 mental disorders, we constructed a collapsed model that treated the corresponding pair of disorders as equivalent. Explicitly, we produced a new binary dummy variable that indicated samples involving either of the two paired mental disorders. For each pair of disorders, the corresponding collapsed model included the dummy variable for that pair of disorders and a separate indicator for both of the other two disorders. This yielded 3 fixed effects. When they were fully interacted with sex of the participant and the gender in the vignette, we obtained 12 fixed effects per collapsed model. Each collapsed model was fit using the full set of samples in the dataset. We conducted a likelihood ratio test to compare each of the 6 collapsed models to the original model with 16 fixed effects. After multiplying the resulting p-values by 6 to account for multiple testing, we assessed whether each of the resulting pairwise comparisons were putatively significant (p < 0.05). For any putatively significant p-values, we performed 100,000 permutations of the outcome vector, again permuting within sub-groups as described above. This yielded robust empirical p-values, which again we multiplied by 6 to account for multiple testing, a Bonferroni correction [31].

Having examined differences in acceptance across mental disorders, we performed one more secondary analysis testing for differences in stigma across the four possible gender combinations within each disorder. Again, we started with the regression model with 16 fixed effects from our primary analysis. For each of the 4 mental disorders, we constructed a collapsed model that treated all four of the gender combinations within that disorder as equivalent. Explicitly, for the mental disorder of interest, we replaced the four variables corresponding to the four gender combinations within that condition with a single new binary dummy variable encoding the disorder of interest. Hence, we obtained 13 fixed effects per collapsed model. Each collapsed model was fit using the full set of samples in the dataset. We conducted a likelihood ratio test to compare each of the 4 collapsed models to the original model with 16 fixed effects. After multiplying the resulting p-values by 4 to account for multiple testing, we assessed whether each of these corrected p-values were putatively significant (p < 0.05). For any putatively significant p-values, we performed 100,000 permutations of the outcome vector, again permuting within sub-groups as described above. This yielded robust empirical p-values, which again we multiplied by 4 to account for multiple testing.

All analyses were conducted in R [32]. The R package, “lme4,” was used for fitting and testing all random effects models [33].

## Results

**Fig 1:**
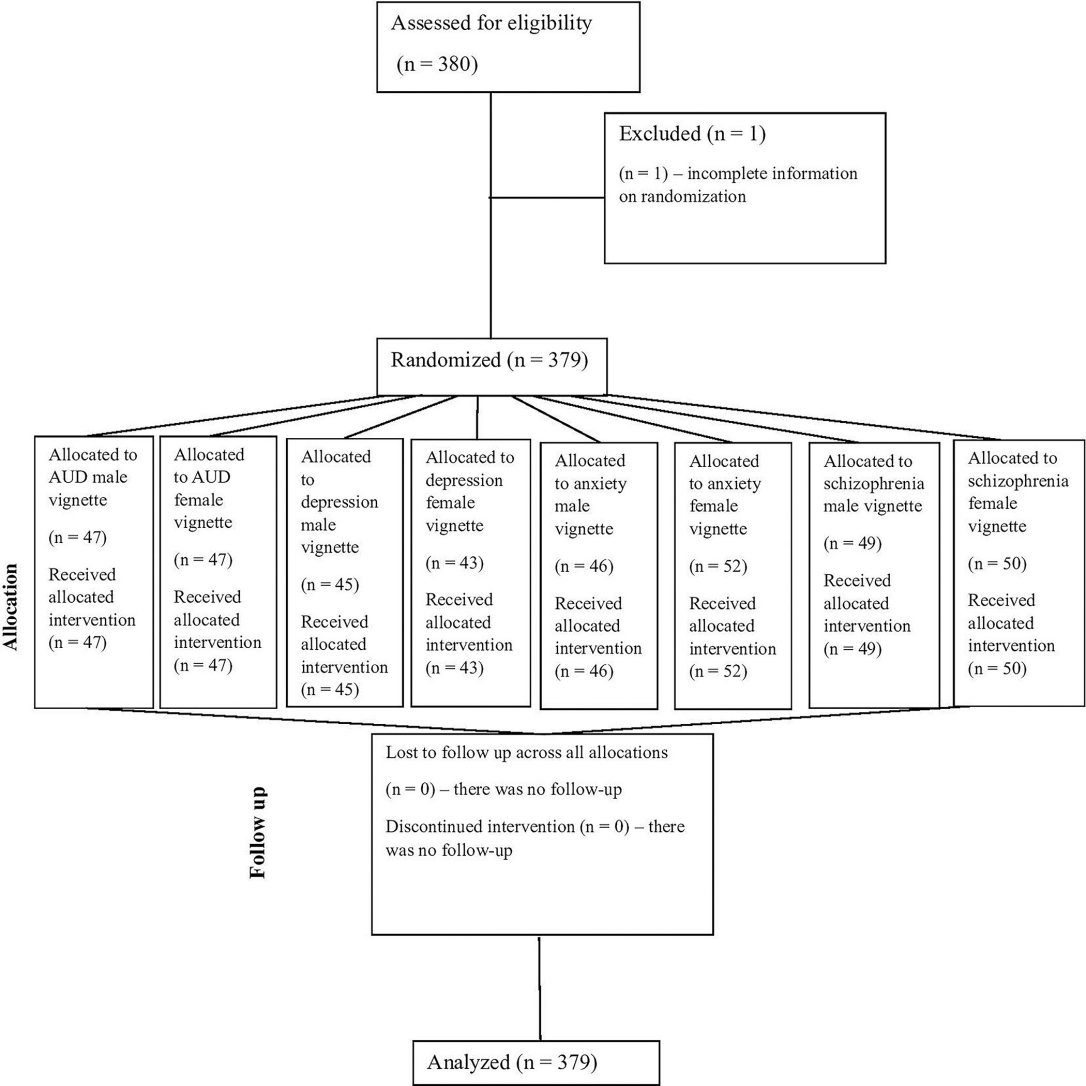
CONSORT diagram showing flow of participants through each stage of the trial.

**Fig 2:**
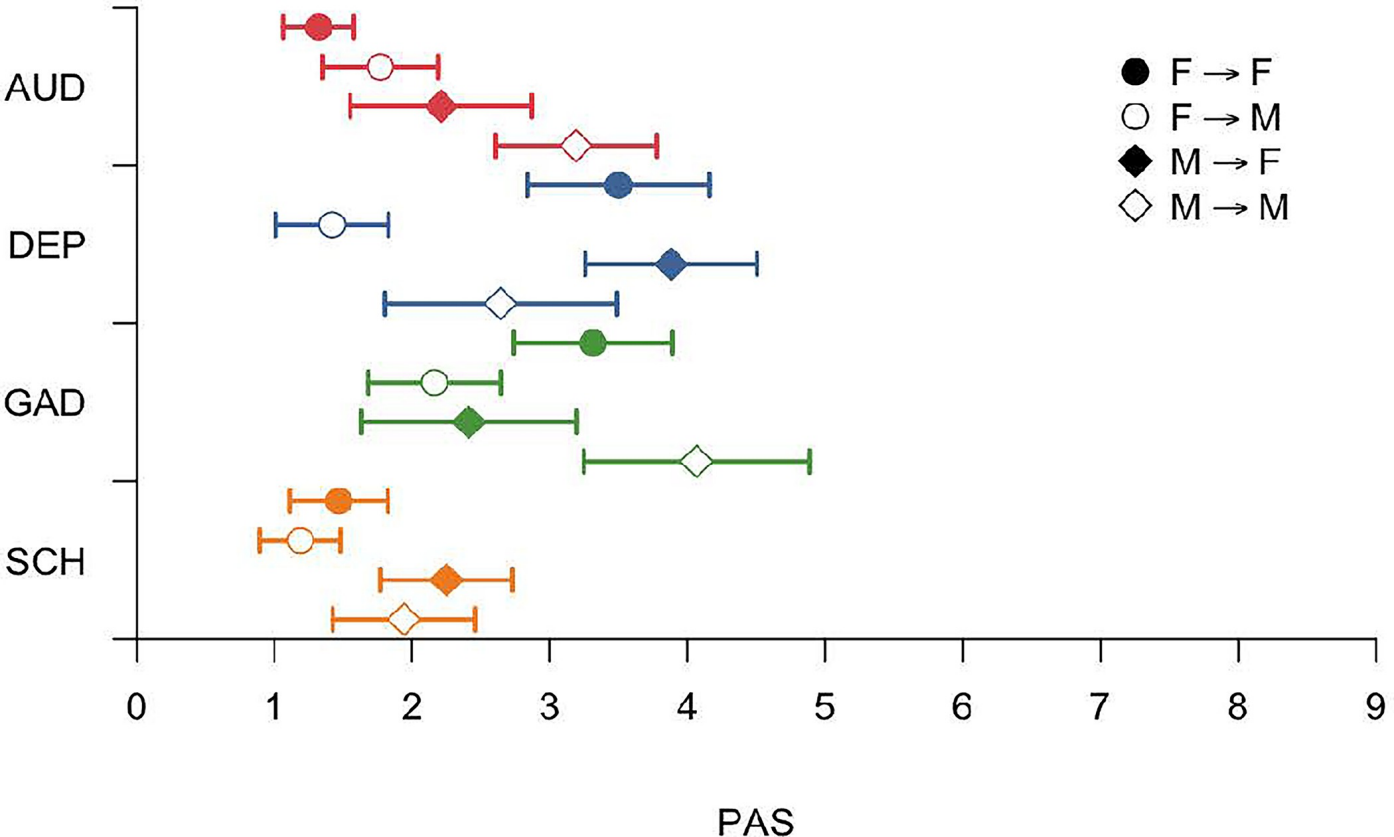
Personal acceptance scale by condition, gender of vignette, and sex of participant means and standard errors esimated under random effects model accounting for village effects. Filled circles denote female participants hearing a female vignette. Hollow circles denote female participants hearing a male vingnette. Filled diamonds denote male participants hearing a male vignette. Hollow diamonds denote male participants hearing a female vignette. See Table 2 for a table of the means and standard errors plotted here.

Of the 380 participants approached, all were included in the study. Data from 1 participant was excluded from the analysis, due to missing data (Fig 1). Participants were identified and interviewed from July 2023 to September 2023. Across vignette groups, there were no significant differences in the distributions of sex, age, and other demographic factors, suggesting successful randomization (Table 1). Fig 2 displays the mean acceptance of PAS estimated across mental disorders and gender combinations by our random effects model accounting for village effects; Table 2 presents these data numerically. The corresponding BAS mean estimates are presented in Appendices C and D. After accounting for multiple testing and model-misspecification via permutation, significant differences in levels of stigma among the four conditions were found for PAS (Bonferroni-adjusted, empirical p-value: 0.00235) and BAS (Bonferroni-adjusted, empirical p-value 0.04757).

**Table 1:** Descriptive statistics.

	AUD		DEP		GAD			SCH		Overall	
Vignette Gender Assignment	F (N = 47)	M (N = 47)	F (N = 43)	M (N = 45)	F (N = 52)	M (N = 46)	F (N = 50)		M (N = 49)	F (N = 192)	M (N = 187)	P-value
**Sex**												
F	28 (59.6%)	26 (55.3%)	26 (60.5%)	31 (68.9%)	35 (67.3%)	31 (67.4%)		30 (60.0%)	32 (65.3%)	119 (62.0%)	120 (64.2%)	0.941
M	19 (40.4%)	21 (44.7%)	17 (39.5%)	14 (31.1%)	17 (32.7%)	15 (32.6%)		20 (40.0%)	17 (34.7%)	73 (38.0%)	67 (35.8%)	
**Age**												
Mean (SD)	35.5 (11.1)	37.4 (13.9)	39.7 (13.7)	37.5 (14.6)	34.8 (12.5)	36.3 (12.4)		38.7 (13.2)	35.4 (10.8)	37.1 (12.7)	36.6 (12.9)	0.79
Median [Min, Max]	32.0 [21.0, 61.0]	35.0 [18.0, 71.0]	37.0 [19.0, 82.0]	33.0 [19.0, 80.0]	32.0 [18.0, 67.0]	33.0 [21.0, 70.0]		36.5 [20.0, 79.0]	35.0 [18.0, 66.0]	35.0 [18.0, 82.0]	35.0 [18.0, 80.0]	
Missing	0 (0%)	1 (2.1%)	0 (0%)	0 (0%)	0 (0%)	1 (2.2%)		0 (0%)	0 (0%)	0 (0%)	2 (1.1%)	
**Marital Status**												
Married or Cohabiting	46 (97.9%)	40 (85.1%)	38 (88.4%)	39 (86.7%)	40 (76.9%)	41 (89.1%)		44 (88.0%)	45 (91.8%)	168 (87.5%)	165 (88.2%)	0.721
Widowed	1 (2.1%)	1 (2.1%)	2 (4.7%)	3 (6.7%)	5 (9.6%)	2 (4.3%)		2 (4.0%)	0 (0%)	10 (5.2%)	6 (3.2%)	
Separated	0 (0%)	2 (4.3%)	3 (7.0%)	1 (2.2%)	3 (5.8%)	1 (2.2%)		1 (2.0%)	2 (4.1%)	7 (3.6%)	6 (3.2%)	
Single & Never Married	0 (0%)	4 (8.5%)	0 (0%)	2 (4.4%)	4 (7.7%)	2 (4.3%)		3 (6.0%)	2 (4.1%)	7 (3.6%)	10 (5.3%)	
**Religion**												
Anglican	14 (29.8%)	17 (36.2%)	17 (39.5%)	15 (33.3%)	17 (32.7%)	14 (30.4%)		14 (28.0%)	16 (32.7%)	62 (32.3%)	62 (33.2%)	0.804
Catholic	9 (19.1%)	4 (8.5%)	8 (18.6%)	5 (11.1%)	11 (21.2%)	8 (17.4%)		13 (26.0%)	14 (28.6%)	41 (21.4%)	31 (16.6%)	
Muslim	12 (25.5%)	13 (27.7%)	7 (16.3%)	13 (28.9%)	18 (34.6%)	15 (32.6%)		11 (22.0%)	8 (16.3%)	48 (25.0%)	49 (26.2%)	
Other (specify)	5 (10.6%)	2 (4.3%)	1 (2.3%)	2 (4.4%)	1 (1.9%)	1 (2.2%)		1 (2.0%)	2 (4.1%)	8 (4.2%)	7 (3.7%)	
Pentecostal	7 (14.9%)	11 (23.4%)	10 (23.3%)	10 (22.2%)	5 (9.6%)	8 (17.4%)		11 (22.0%)	9 (18.4%)	33 (17.2%)	38 (20.3%)	
**Occupation**												
Other (Type)	3 (6.4%)	2 (4.3%)	5 (11.6%)	1 (2.2%)	3 (5.8%)	5 (10.9%)		3 (6.0%)	3 (6.1%)	14 (7.3%)	11 (5.9%)	0.522
Peasant farmer	44 (93.6%)	45 (95.7%)	37 (86.0%)	44 (97.8%)	49 (94.2%)	39 (84.8%)		47 (94.0%)	46 (93.9%)	177 (92.2%)	174 (93.0%)	
Shop owner	0 (0%)	0 (0%)	1 (2.3%)	0 (0%)	0 (0%)	2 (4.3%)		0 (0%)	0 (0%)	1 (0.5%)	2 (1.1%)	
**Education**												
Complete Primary	7 (14.9%)	8 (17.0%)	5 (11.6%)	8 (17.8%)	7 (13.5%)	6 (13.0%)		8 (16.0%)	7 (14.3%)	27 (14.1%)	29 (15.5%)	0.972
Incomplete Primary	29 (61.7%)	17 (36.2%)	18 (41.9%)	17 (37.8%)	24 (46.2%)	24 (52.2%)		21 (42.0%)	24 (49.0%)	92 (47.9%)	82 (43.9%)	
Never Attended	5 (10.6%)	12 (25.5%)	9 (20.9%)	10 (22.2%)	11 (21.2%)	9 (19.6%)		10 (20.0%)	9 (18.4%)	35 (18.2%)	40 (21.4%)	
O-Level	6 (12.8%)	10 (21.3%)	9 (20.9%)	10 (22.2%)	10 (19.2%)	7 (15.2%)		8 (16.0%)	9 (18.4%)	33 (17.2%)	36 (19.3%)	
A-Level	0 (0%)	0 (0%)	1 (2.3%)	0 (0%)	0 (0%)	0 (0%)		1 (2.0%)	0 (0%)	2 (1.0%)	0 (0%)	
Vocational Training	0 (0%)	0 (0%)	1 (2.3%)	0 (0%)	0 (0%)	0 (0%)		1 (2.0%)	0 (0%)	2 (1.0%)	0 (0%)	
Post-Secondary	0 (0%)	0 (0%)	0 (0%)	0 (0%)	0 (0%)	0 (0%)		1 (2.0%)	0 (0%)	1 (0.5%)	0 (0%)	
**Village**												
Bukutula/Bukutula LC1	3 (6.4%)	3 (6.4%)	2 (4.7%)	1 (2.2%)	4 (7.7%)	1 (2.2%)		3 (6.0%)	4 (8.2%)	12 (6.3%)	9 (4.8%)	1
Bukutula/Bulagala	2 (4.3%)	2 (4.3%)	1 (2.3%)	2 (4.4%)	2 (3.8%)	1 (2.2%)		2 (4.0%)	2 (4.1%)	7 (3.6%)	7 (3.7%)	
Bukutula/Bunangwe LC	2 (4.3%)	3 (6.4%)	4 (9.3%)	2 (4.4%)	1 (1.9%)	3 (6.5%)		2 (4.0%)	3 (6.1%)	9 (4.7%)	11 (5.9%)	
Bukutula/Masaba	2 (4.3%)	4 (8.5%)	2 (4.7%)	2 (4.4%)	4 (7.7%)	3 (6.5%)		2 (4.0%)	2 (4.1%)	10 (5.2%)	11 (5.9%)	
Gumpi/Busikwe A	2 (4.3%)	1 (2.1%)	2 (4.7%)	3 (6.7%)	2 (3.8%)	3 (6.5%)		3 (6.0%)	4 (8.2%)	9 (4.7%)	11 (5.9%)	
Gumpi/Gabula	2 (4.3%)	3 (6.4%)	1 (2.3%)	3 (6.7%)	4 (7.7%)	2 (4.3%)		2 (4.0%)	3 (6.1%)	9 (4.7%)	11 (5.9%)	
Igalaza/Bulondo	3 (6.4%)	2 (4.3%)	3 (7.0%)	3 (6.7%)	3 (5.8%)	4 (8.7%)		2 (4.0%)	0 (0%)	11 (5.7%)	9 (4.8%)	
Igalaza/Buteira	2 (4.3%)	2 (4.3%)	3 (7.0%)	3 (6.7%)	1 (1.9%)	2 (4.3%)		1 (2.0%)	5 (10.2%)	7 (3.6%)	12 (6.4%)	
Igalaza/Igalaza	2 (4.3%)	3 (6.4%)	5 (11.6%)	0 (0%)	4 (7.7%)	3 (6.5%)		3 (6.0%)	1 (2.0%)	14 (7.3%)	7 (3.7%)	
Igalaza/Ngereka	3 (6.4%)	3 (6.4%)	1 (2.3%)	3 (6.7%)	2 (3.8%)	3 (6.5%)		2 (4.0%)	3 (6.1%)	8 (4.2%)	12 (6.4%)	
Kabukye/Bunangwe	2 (4.3%)	3 (6.4%)	2 (4.7%)	2 (4.4%)	2 (3.8%)	2 (4.3%)		4 (8.0%)	3 (6.1%)	10 (5.2%)	10 (5.3%)	
Kagulu/Gabona	3 (6.4%)	3 (6.4%)	2 (4.7%)	4 (8.9%)	1 (1.9%)	1 (2.2%)		3 (6.0%)	1 (2.0%)	9 (4.7%)	9 (4.8%)	
Kagulu/Kibila Kyeza	3 (6.4%)	1 (2.1%)	1 (2.3%)	2 (4.4%)	2 (3.8%)	1 (2.2%)		2 (4.0%)	2 (4.1%)	8 (4.2%)	6 (3.2%)	
Kagulu/Nalina	2 (4.3%)	2 (4.3%)	3 (7.0%)	3 (6.7%)	4 (7.7%)	1 (2.2%)		3 (6.0%)	2 (4.1%)	12 (6.3%)	8 (4.3%)	
Kimbaya/Bulangira B	2 (4.3%)	2 (4.3%)	1 (2.3%)	2 (4.4%)	3 (5.8%)	3 (6.5%)		4 (8.0%)	3 (6.1%)	10 (5.2%)	10 (5.3%)	
Kirimwa/Bukongoro	2 (4.3%)	1 (2.1%)	2 (4.7%)	3 (6.7%)	2 (3.8%)	3 (6.5%)		3 (6.0%)	4 (8.2%)	9 (4.7%)	11 (5.9%)	
Kirimwa/Kinatebe	3 (6.4%)	1 (2.1%)	1 (2.3%)	3 (6.7%)	3 (5.8%)	2 (4.3%)		2 (4.0%)	2 (4.1%)	9 (4.7%)	8 (4.3%)	
Kirimwa/Kiramugira	2 (4.3%)	4 (8.5%)	1 (2.3%)	1 (2.2%)	4 (7.7%)	3 (6.5%)		2 (4.0%)	3 (6.1%)	9 (4.7%)	11 (5.9%)	
Nsomba/Bugembe	5 (10.6%)	3 (6.4%)	4 (9.3%)	1 (2.2%)	2 (3.8%)	2 (4.3%)		3 (6.0%)	0 (0%)	14 (7.3%)	6 (3.2%)	
Bukutula/Nakawuna	0 (0%)	1 (2.1%)	2 (4.7%)	2 (4.4%)	2 (3.8%)	3 (6.5%)		2 (4.0%)	2 (4.1%)	6 (3.1%)	8 (4.3%)	
**Household Size**												
Mean (SD)	3.02 (1.75)	3.34 (2.42)	4.16 (2.67)	3.36 (2.46)	2.98 (1.59)	2.96 (2.46)		2.88 (1.59)	2.82 (1.63)	3.23 (1.97)	3.11 (2.25)	0.146
Median [Min, Max]	2.00 [1.00, 8.00]	2.00 [1.00, 11.0]	3.00 [1.00, 10.0]	2.00 [2.00, 15.0]	2.00 [1.00, 8.00]	2.00 [1.00, 16.0]		2.00 [0, 8.00]	2.00 [1.00, 8.00]	2.00 [0, 10.0]	2.00 [1.00, 16.0]	
**BAS**												
Mean (SD)	3.36 (1.31)	3.34 (1.55)	3.81 (1.45)	3.20 (1.29)	3.83 (1.71)	3.98 (1.86)		3.26 (1.23)	3.29 (1.17)	3.56 (1.45)	3.45 (1.51)	0.13
Median [Min, Max]	3.00 [0, 6.00]	3.00 [1.00, 8.00]	4.00 [0, 7.00]	3.00 [0, 7.00]	4.00 [0, 9.00]	4.00 [0, 9.00]		3.00 [0, 7.00]	3.00 [2.00, 7.00]	3.00 [0, 9.00]	3.00 [0, 9.00]	
**PAS**												
Mean (SD)	1.68 (2.13)	2.40 (2.47)	3.65 (3.05)	1.80 (2.61)	3.02 (3.34)	2.78 (2.96)		1.78 (2.05)	1.45 (1.85)	2.51 (2.80)	2.10 (2.53)	0.0023
Median [Min, Max]	1.00 [0, 8.00]	1.00 [0, 8.00]	3.00 [0, 9.00]	0 [0, 9.00]	1.00 [0, 9.00]	2.00 [0, 9.00]		1.00 [0, 9.00]	1.00 [0, 8.00]	1.00 [0, 9.00]	1.00 [0, 9.00]	

**Table 2:** Table of means for the personal acceptance scale by condition, gender of vignette, and sex of participant–means and standard errors estimated under random effects model accounting for village effects.

Condition	Sex of Participant	Gender of Vignette	Mean	SE
AUD	F	F	1.301411	0.4794370
AUD	F	M	1.768187	0.4964552
AUD	M	F	2.231659	0.5797616
AUD	M	M	3.148529	0.5530488
DEP	F	F	3.504249	0.4966921
DEP	F	M	1.423370	0.4553088
DEP	M	F	3.885468	0.6130191
DEP	M	M	2.642271	0.6746283
GAD	F	F	3.330578	0.4304355
GAD	F	M	2.190806	0.4562457
GAD	M	F	2.371943	0.6148696
GAD	M	M	4.038042	0.6539972
SCH	F	F	1.471217	0.4632611
SCH	F	M	1.206004	0.4498749
SCH	M	F	2.199728	0.5657176
SCH	M	M	1.927122	0.6157802

See Fig 1 for a visual display of these data.

In our secondary pairwise analysis, we found that acceptance differed between anxiety and schizophrenia according to PAS (Bonferroni-adjusted, empirical p-value 0.008) and BAS (Bonferroni-adjusted, empirical p-value 0.007). Visual inspection of Figs 2 and S1 suggests that individuals with anxiety are more accepted than individuals with schizophrenia. None of the other pairwise comparisons reached statistical significance for PAS or BAS (Appendices E and F).

In our final secondary analysis examining differences in acceptance across gender combinations within mental disorders, we found that PAS varied across gender combinations for depression (Bonferroni-adjusted, empirical p-value 0.017). In the context of Fig 2, we interpret this result as a reflection of personal acceptance being higher for women with depression than men with depression. Significant gender effects were not observed for any of the other mental disorders on the PAS (S4 Table) or for any of the four disorders on the BAS (S5 Table).

## Discussion

In this population-based, experimental vignette study conducted in a rural region of eastern Uganda, we found significant differences in levels of stigma attached to depression, anxiety, alcohol use disorder, and schizophrenia. The differences persisted after adjusting for the sex of the respondent and the sex of the individual depicted in the vignette. Schizophrenia was least accepted, or most highly stigmatized, among the four conditions. In pairwise comparisons after adjusting for multiple testing, people with anxiety disorder appeared to be more accepted than people with schizophrenia.

The finding that anxiety is less stigmatized than schizophrenia aligns with existing literature from HICs [30]. In HICs, research indicates that mental illnesses conforming to gender-based stereotypes are more likely to face stigma, although other studies have shown no association [16,17]. It is possible that with a greater sample size, our study would have been powered to detect differences in stigmatizing attitudes between depression and alcohol use disorder and between depression and schizophrenia, given that pairwise comparisons without the Bonferroni correction demonstrated significant differences. This would be in line with previous findings that alcohol use disorder is stigmatized at a similar level as schizophrenia, and that schizophrenia and alcohol use disorder are more stigmatized than other mental illnesses [34].

Our measurement of differential levels of stigma based on the specific mental disorder, adjusting for sex of the respondent and the sex of the individual depicted in the vignette, provided us with a unique ability to assess mental illness stigma in a general population sample from a low-income country. Our use of a general population sample is a strength given that community and family support are critical predictors of mental health outcomes, particularly in LMICs where mental healthcare may be more difficult to access and where formal safety nets may be more limited [1,35]. Most studies on mental illness stigma have been in HICs, whereas 80% of the burden of mental illness is in LMICs, and stigma appears to be an even stronger barrier to treatment access in low resource settings [36]. The Stigma in Global Context–Mental Health Study assessed stigmatizing attitudes toward people with depression and schizophrenia but did not include any low-income African countries [37].

The random assignment of exposure to vignettes strengthens our confidence that the observed differences were not the result of selection bias. The vignette approach towards describing mental illness has been utilized in some studies investigating mental illness stigma, but not universally [38]. Participants in the Stigma in Global Context–Mental Health Study were randomly assigned to vignettes about depression and schizophrenia [37]. Rasmussen et al. conducted an experimental vignette study with a population-based sample in Uganda to assess the extent to which portrayals of successful treatment could attenuate stigmatizing attitudes toward people with depression, schizophrenia, and bipolar disorder [21]. Our study further assesses stigmatizing attitudes toward people with generalized anxiety disorder and alcohol use disorder. Our vignettes were informed by the DSM-5 and modified by Ugandan experts to generate locally acceptable and understandable language and communication. A psychiatrist developed the criteria for the vignettes per DSM-5, and a local team developed the vignettes per the criteria in a manner that is relatable to the local audience.

Our data could suggest that mental disorders with behavioral manifestations deviating from gender norms, such as men with depression, face greater stigmatization. [Fig 2] This finding is potentially consistent with population-based epidemiological studies from Uganda suggesting that depression is much more prevalent among women than men [39,40]. These results could also align with previous studies demonstrating the widespread stigmatization of depression among men across different cultures, often attributed to perceptions of “weakness in character [41,42].” However, there needs to be further studies to investigate this hypothesis, as it was not the primary outcome of our study.

A limitation of the study is that although the vignettes were translated from Runyankore to Lusoga [21], modified by experts to ensure linguistic and cultural appropriateness to the Busogan context, and then back-translated to English to verify that they appropriately represented psychiatric concepts similar to how they would be understood in the U.S., such procedures can inadvertently affect the properties of the tools. Subtle shifts in meaning could influence respondent understanding and responses, possibly affecting the nature of the responses elicited. However, the stigma outcome scales have demonstrated good reliability and construct validity in the Busogan context [24,25] and elsewhere in Africa [22]. Other limitations of the study include that the study design relied upon responses to a questionnaire and was therefore vulnerable to social desirability bias. One might expect that social desirability bias affected all conditions similarly, but it is also possible that certain conditions were associated with greater social desirability bias than others and that this differential social desirability bias could have affected the findings. Observing behavior, instead of relying on a questionnaire, would have potentially addressed this problem. Additionally, the study sampled a limited geographical area and cannot be directly generalized to other areas.

Stigma surrounding mental illness continues to be a neglected issue in LMICs, with limited exploration into its nuanced aspects taking specific disorders and gender into consideration [43]. This study has contributed valuable insights by examining illness stigma in LMICs, specifically by dissecting stigma levels based on the gender of the individual affected, the participant’s sex, and the specific mental health condition involved. To ascertain the broader applicability of these findings, further research is essential in diverse LMIC contexts. Further research could also investigate how gender affects attitudes towards specific mental illnesses. The data from this study can serve as a foundation for targeted anti-stigma interventions, highlighting the importance of considering specific mental disorders and gender norms in shaping these stigmatizing attitudes.

## Supporting information

S1 Checklist CONSORT checklist. (DOCX)

S1 Text Sample vignette depicting woman with depression. (DOCX)

S2 Text Items in the broad acceptance scale and the personal acceptance scale. (DOCX)

S1 Fig Broad acceptance scale by condition, gender of vignette, and sex of participant–means and standard errors estimated under random effects model accounting for village effects. (TIFF)

S1 Table Table of means for the broad acceptance scale by condition, gender of vignette, and sex of participant–means and standard errors estimated under random effects model accounting for village effects. (DOCX)

S2 Table P-values for each likelihood ratio test testing for differences in PAS between pairs of diagnostic disorders. P-values were multiplied by 6 for Bonferroni adjustment for multiple comparisons. For the four p-values that were nominally significant, we performed 100,000 permutations and provided the corresponding Bonferroni adjusted, empirical p-values in parentheses.
(DOCX)

S3 Table P-values for each likelihood ratio test testing for differences in BAS between pairs of diagnostic disorders. P-values were multiplied by 6 for Bonferroni adjustment for multiple comparisons. For the four p-values that were nominally significant, we performed 100,000 permutations and provided the corresponding Bonferroni adjusted, empirical p-values in parentheses.
(DOCX)

S4 Table P-values for each likelihood ratio test testing for differences in PAS across gender combinations within each diagnostic disorder. P-values were multiplied by 4 for Bonferroni adjustment for multiple comparisons. For the one p-value that was nominally significant, we performed 100,000 permutations and provided the corresponding Bonferroni adjusted, empirical p-value in parentheses.
(DOCX)

S5 Table P-values for each likelihood ratio test testing for differences in BAS across gender combinations within each diagnostic disorder. P-values were multiplied by 4 for Bonferroni adjustment for multiple comparisons. None of the p-values were nominally significant.
(DOCX)
