# Supplementary material for: Differences in mental illness stigma by disorder and gender: Population-based vignette randomized experiment in rural Uganda
Source: PLOS Ment Health. 2024 Jun 21;1(1):e0000069. doi: 10.1371/journal.pmen.0000069 (PMC11345708; doi:10.1371/journal.pmen.0000069)
Supplement: S1 Text — (DOCX) [file pmen.0000069.s002.docx]

**Sample vignette depicting woman with depression**

Imagine there is a young Musoga woman who has completed primary school. She has never taken alcohol or drugs, nor has she ever contracted any major medical illness such as HIV. She enjoys working in the family’s maize garden and taking part in various activities in the community. Sometimes she might think too much about things and be sad, but this only happened twice and only for a short time. She has experienced the usual ups and downs of life, but managed to get through the challenges she has faced.

One day, she started to feel very sad. She began to wake up in the morning with a flat, heavy feeling that remained with her all day long. She lost her drive to participate in her usual work and community activities and soon began spending most of the day alone in her room. In fact, nothing seemed to give her pleasure. Even when good things happened, like when the family had a good harvest, she could not feel happy. She began to find it extremely difficult to accomplish anything, and it seemed like life was such a burden. She lacked energy all the time and felt tired, but when night came she found it a struggle to sleep. She found that she could not concentrate on things and felt like she was thinking too much. She felt like her life was worthless. She even thought about taking her own life.
