# Supplementary material for: Differences in mental illness stigma by disorder and gender: Population-based vignette randomized experiment in rural Uganda
Source: PLOS Ment Health. 2024 Jun 21;1(1):e0000069. doi: 10.1371/journal.pmen.0000069 (PMC11345708; doi:10.1371/journal.pmen.0000069)
Supplement: S2 Text — (DOCX) [file pmen.0000069.s003.docx]

**Items in the Broad Acceptance Scale and the Personal Acceptance Scale**

| Broad Acceptance Scale | Personal Acceptance Scale |
| --- | --- |
| ^a^ This person should not be given any responsibility | ^a^ I am afraid of people such as the one depicted |
| ^a^ This person is a burden on society | ^a^ Would you object to having this person living in your neighborhood? |
| This person should have the same rights to a job as anyone else | Would you be willing to work with this person? |
| We have a responsibility to provide the best possible care for this person | Would you invite this person into your home? |
| ^a^ Increased spending on services for people like this is a waste of money | ^a^ I would not want to live next door to this person |
| We need to adopt a far more tolerant attitude toward people like this in our society | ^a^ It is frightening to think of this person being a neighbor |
| ^a^ People with the condition depicted are a public nuisance | Would you have casual conversations with this person |
| ^a^ This person should be excluded from taking public office | I would let this person watch my child |
| This person can work in regular jobs | ^a^ Would you avoid conversations with this person if they are neighbors? |

## **Notes:** ^a^ denotes items that were reverse coded.
