# Supplementary figures and images for: Differences in mental illness stigma by disorder and gender: Population-based vignette randomized experiment in rural Uganda
Source: PLOS Ment Health. 2024 Jun 21;1(1):e0000069. doi: 10.1371/journal.pmen.0000069 (PMC11345708; doi:10.1371/journal.pmen.0000069)

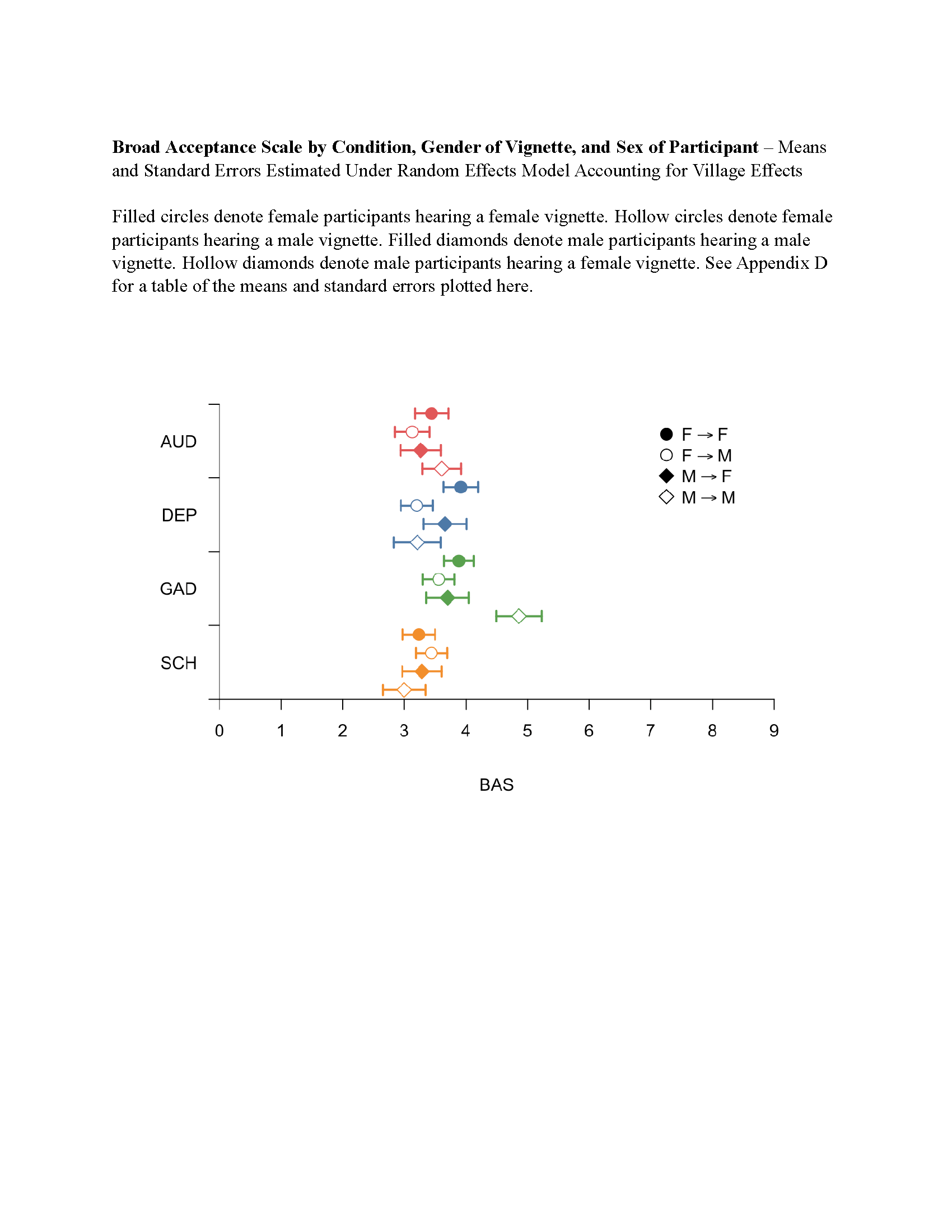

Supplement: S1 Fig — (TIFF) [file pmen.0000069.s004.tiff]
