# Supplementary material for: Differences in mental illness stigma by disorder and gender: Population-based vignette randomized experiment in rural Uganda
Source: PLOS Ment Health. 2024 Jun 21;1(1):e0000069. doi: 10.1371/journal.pmen.0000069 (PMC11345708; doi:10.1371/journal.pmen.0000069)
Supplement: S1 Table — (DOCX) [file pmen.0000069.s005.docx]

## **Table of Means for the Broad Acceptance Scale by Condition, Gender of Vignette, and Sex of Participant** – Means and Standard Errors Estimated Under Random Effects Model Accounting for Village Effects.

| **Condition** | **Sex of Participant** | **Gender of Vignette** | **Mean** | **SE** |
| --- | --- | --- | --- | --- |
| AUD | F | F | 3.441946 | 0.2708271 |
| AUD | F | M | 3.126647 | 0.2806469 |
| AUD | M | F | 3.266091 | 0.3279712 |
| AUD | M | M | 3.607200 | 0.3125652 |
| DEP | F | F | 3.915547 | 0.2807396 |
| DEP | F | M | 3.201287 | 0.2572384 |
| DEP | M | F | 3.659433 | 0.3467872 |
| DEP | M | M | 3.210343 | 0.3818265 |
| GAD | F | F | 3.885227 | 0.2428190 |
| GAD | F | M | 3.557535 | 0.2576019 |
| GAD | M | F | 3.701879 | 0.3475043 |
| GAD | M | M | 4.860028 | 0.3697310 |
| SCH | F | F | 3.236203 | 0.2616631 |
| SCH | F | M | 3.439616 | 0.2538568 |
| SCH | M | F | 3.285993 | 0.3199113 |
| SCH | M | M | 2.997391 | 0.3478589 |
