# Supplementary material for: Differences in mental illness stigma by disorder and gender: Population-based vignette randomized experiment in rural Uganda
Source: PLOS Ment Health. 2024 Jun 21;1(1):e0000069. doi: 10.1371/journal.pmen.0000069 (PMC11345708; doi:10.1371/journal.pmen.0000069)
Supplement: S2 Table — P-values were multiplied by 6 for Bonferroni adjustment for multiple comparisons. For the four p-values that were nominally significant, we performed 100,000 permutations and provided the corresponding Bonferroni adjusted, empirical p-values in parentheses. (DOCX) [file pmen.0000069.s006.docx]

## **P-values for each likelihood ratio test testing for differences in PAS between pairs of diagnostic disorders.** P-values were multiplied by 6 for Bonferroni adjustment for multiple comparisons. For the four p-values that were nominally significant, we performed 100,000 permutations and provided the corresponding Bonferroni adjusted, empirical p-values in parentheses.

|  | | | | |
| --- | --- | --- | --- | --- |
|  | **AUD** | **DEP** | **GAD** | **SCH** |
| AUD |  | 0.034 (0.115) | 0.132 | 1 |
| DEP |  |  | 0.887 | 0.049 (0.089) |
| GAD |  |  |  | 0.014 (0.008) |
| SCH |  |  |  |  |
