# Supplementary material for: Differences in mental illness stigma by disorder and gender: Population-based vignette randomized experiment in rural Uganda
Source: PLOS Ment Health. 2024 Jun 21;1(1):e0000069. doi: 10.1371/journal.pmen.0000069 (PMC11345708; doi:10.1371/journal.pmen.0000069)
Supplement: S4 Table — P-values were multiplied by 4 for Bonferroni adjustment for multiple comparisons. For the one p-value that was nominally significant, we performed 100,000 permutations and provided the corresponding Bonferroni adjusted, empirical p-value in parentheses. (DOCX) [file pmen.0000069.s008.docx]

**P-values for each likelihood ratio test testing for differences in PAS across gender combinations within each diagnostic disorder.** P-values were multiplied by 4 for Bonferroni adjustment for multiple comparisons. For the one p-value that was nominally significant, we performed 100,000 permutations and provided the corresponding Bonferroni adjusted, empirical p-value in parentheses.

|  | | | |
| --- | --- | --- | --- |
| **AUD** | **DEP** | **GAD** | **SCH** |
| 0.307 | 0.011 (0.017) | 0.261 | 1 |

|  |
| --- |
