# Supplementary material for: Differences in mental illness stigma by disorder and gender: Population-based vignette randomized experiment in rural Uganda
Source: PLOS Ment Health. 2024 Jun 21;1(1):e0000069. doi: 10.1371/journal.pmen.0000069 (PMC11345708; doi:10.1371/journal.pmen.0000069)
Supplement: S5 Table — P-values were multiplied by 4 for Bonferroni adjustment for multiple comparisons. None of the p-values were nominally significant. (DOCX) [file pmen.0000069.s009.docx]

**P-values for each likelihood ratio test testing for differences in BAS across gender combinations within each diagnostic disorder.** P-values were multiplied by 4 for Bonferroni adjustment for multiple comparisons. None of the p-values were nominally significant.

|  | | | |
| --- | --- | --- | --- |
| **AUD** | **DEP** | **GAD** | **SCH** |
| 1 | 0.905 | 0.130 | 1 |
